# Infertility and Risk of Autism Spectrum Disorder in Children

**DOI:** 10.1001/jamanetworkopen.2023.43954

**Published:** 2023-11-20

**Authors:** Maria P. Velez, Natalie Dayan, Jonas Shellenberger, Jessica Pudwell, Dia Kapoor, Simone N. Vigod, Joel G. Ray

**Affiliations:** 1 Department of Obstetrics and Gynaecology, Queen’s University, Kingston, Ontario, Canada; 2 ICES, Toronto, Ontario, Canada; 3 Department of Medicine, Obstetrics and Gynaecology, and Research Institute, McGill University Health Centre, Montreal, Quebec, Canada; 4 Department of Epidemiology, Biostatistics and Occupational Health, McGill University, Montreal, Quebec, Canada; 5 Department of Psychiatry, Temerty Faculty of Medicine, University of Toronto, Toronto, Ontario, Canada; 6 Women’s College Hospital and Women’s College Research Institute, Toronto, Ontario, Canada; 7 Department of Medicine and Obstetrics and Gynaecology, Temerty Faculty of Medicine, St Michael’s Hospital, University of Toronto, Toronto, Ontario, Canada

## Abstract

**Importance:**

Previous studies on the risk of childhood autism spectrum disorder (ASD) following fertility treatment did not account for the infertility itself or the mediating effect of obstetrical and neonatal factors.

**Objective:**

To assess the association between infertility and its treatments on the risk of ASD and the mediating effect of selected adverse pregnancy outcomes on that association.

**Design, Setting, and Participants:**

This was a population-based cohort study in Ontario, Canada. Participants were all singleton and multifetal live births at 24 or more weeks’ gestation from 2006 to 2018. Data were analyzed from October 2022 to October 2023.

**Exposures:**

The exposure was mode of conception, namely, (1) unassisted conception, (2) infertility without fertility treatment (ie, subfertility), (3) ovulation induction (OI) or intrauterine insemination (IUI), or (4) in vitro fertilization (IVF) or intracytoplasmic sperm injection (ICSI).

**Main Outcome and Measures:**

The study outcome was a diagnosis of ASD at age 18 months or older. Cox regression models generated hazard ratios (HR) adjusted for maternal and infant characteristics. Mediation analysis further accounted for the separate effect of (1) preeclampsia, (2) cesarean birth, (3) multifetal pregnancy, (4) preterm birth at less than 37 weeks, and (5) severe neonatal morbidity.

**Results:**

A total of 1 370 152 children (703 407 male [51.3%]) were included: 1 185 024 (86.5%) with unassisted conception, 141 180 (10.3%) with parental subfertility, 20 429 (1.5%) following OI or IUI, and 23 519 (1.7%) following IVF or ICSI. Individuals with subfertility or fertility treatment were older and resided in higher-income areas; the mean (SD) age of each group was as follows: 30.1 (5.2) years in the unassisted conception group, 33.3 (4.7) years in the subfertility group, 33.1 (4.4) years in the OI or IUI group, and 35.8 (4.9) years in the IVF or ICSI group. The incidence rate of ASD was 1.93 per 1000 person-years among children in the unassisted conception group. Relative to the latter, the adjusted HR for ASD was 1.20 (95% CI, 1.15-1.25) in the subfertility group, 1.21 (95% CI, 1.09-1.34) following OI or IUI, and 1.16 (95% CI, 1.04-1.28) after IVF or ICSI. Obstetrical and neonatal factors appeared to mediate a sizeable proportion of the aforementioned association between mode of conception and ASD risk. For example, following IVF or ICSI, the proportion mediated by cesarean birth was 29%, multifetal pregnancy was 78%, preterm birth was 50%, and severe neonatal morbidity was 25%.

**Conclusions and Relevance:**

In this cohort study, a slightly higher risk of ASD was observed in children born to individuals with infertility, which appears partly mediated by certain obstetrical and neonatal factors. To optimize child neurodevelopment, strategies should further explore these other factors in individuals with infertility, even among those not receiving fertility treatment.

## Key Points

**Question:**

Is there an association between infertility, its treatment, and autism spectrum disorder (ASD) in the child?

**Findings:**

In this cohort study of 1.3 million children from Ontario, Canada, the incidence rate (per 1000 person-years) of ASD was 1.9 among children in the unassisted conception group, 2.5 in the subfertility group, and 2.7 after fertility treatment. There was a slightly higher risk of ASD in children born to individuals with infertility, which may be partly mediated by obstetrical and neonatal factors.

**Meaning:**

These findings suggest broader strategies are needed to decrease adverse pregnancy outcomes in patients with infertility and to optimize child neurodevelopment.

## Teaser

This cohort study evaluates the association between infertility and autism spectrum disorder in children in Canada.

## Introduction

The origin of autism spectrum disorder (ASD) likely involves genetic and environmental factors.^1^ Given that ASD symptoms can be present as early as 18 months of age,^1^ risk factors occurring during the perinatal period are important to consider.^2,3,4,5^ Included among these risk factors is the role of infertility and its treatments, the importance of which is underscored by the fact that 1 in 6 couples receive an infertility diagnosis,^6^ and approximately 10 million infants have been born worldwide after the use of fertility treatments. Fertility treatments range from less invasive (ovulation induction [OI] and intrauterine insemination [IUI]), to more invasive treatments (in vitro fertilization [IVF] and intracytoplasmic sperm injection [ICSI]).^7^

In the pathogenesis of ASD, maternal metabolic and inflammatory factors, as well as offspring epigenetic changes, have each been implicated.^8,9,10,11^ Common maternal metabolic and inflammatory disorders include polycystic ovary syndrome (PCOS),^12^ endometriosis,^13^ and obesity.^14^ Moreover, epigenetic changes have been found in children of women with a prolonged antecedent duration of infertility, and also in those conceived by ICSI, the main indication of which is male infertility.^15^ Possible mechanistic factors for epigenetic changes after IVF or ICSI include the embryo culture media and the manipulation of the oocyte and sperm during ICSI.^15^

Initial studies have reported little to no risk of ASD in children born after OI, IUI, IVF, or ICSI.^16,17,18,19,20,21,22,23,24,25^ In addition, there may be a higher risk of ASD in children born to parents with infertility, but who otherwise did not receive fertility treatment, known as subfertility.^16,26,27^ Although individuals with subfertility and those receiving fertility therapy are at a higher risk of adverse pregnancy outcomes, including preeclampsia, cesarean birth, multiple pregnancy, preterm birth, and severe neonatal morbidity,^28,29,30,31,32,33^ there is limited data about the mediating effect of those factors on the association between mode of conception and ASD. The current study evaluated the association between infertility and fertility treatment and the risk of ASD, while further modeling the mediating effect of adverse pregnancy outcomes.

## Methods

This retrospective, population-based study was conducted using existing linked administrative health data from Ontario, Canada (eTable 1 in Supplement 1). Data were linked using unique maternal and child identifiers and analyzed at ICES,^34^ an independent, nonprofit research institute whose legal status under Ontario’s health information privacy law allows it to collect and analyze health care and demographic data without consent for health system evaluation and improvement. The study followed the Strengthening the Reporting of Observational Studies in Epidemiology (STROBE) reporting guideline for cohort studies and was reviewed for ethical compliance by the Queen’s University Health Sciences and Affiliated Teaching Hospitals research ethics board.

### Data Sources and Study Cohort Creation

Included were all singleton and multiple hospital live births at 24 or more weeks’ gestation, from April 1, 2006, to March 31, 2018, and among individuals aged 18 to 55 years. Setting the year 2018 for the last birth permitted all eligible children to be assessed for ASD at a minimum age of 4 years by June 2022. Additional study databases included those capturing all hospitalizations, emergency department visits, and outpatient visits, as detailed in eTable 1 in Supplement 1. Pregnancy characteristics, including mode of conception, were obtained from the Better Outcomes Registry and Network (BORN) Ontario database and its related Niday Legacy data sets,^35^ which represent approximately 99% of hospital births in Ontario and have been previously validated for completeness and accuracy.^36^ Excluded were surrogate pregnancies, a pregnancy ending in an induced abortion, a child death before 18 months of age, and those with incomplete records (Figure).

**Figure.**
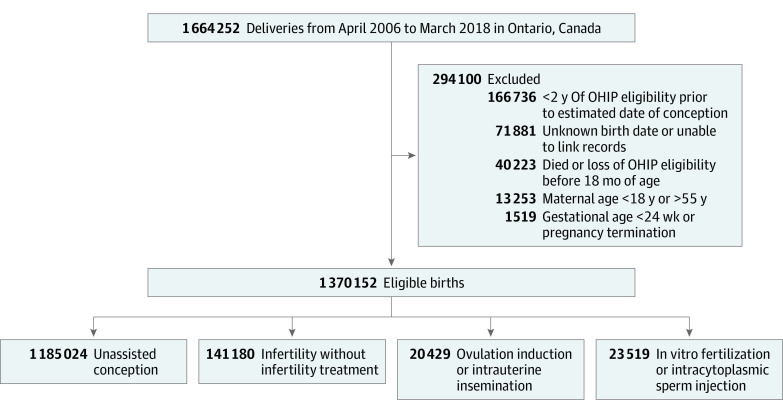
Cohort Creation OHIP indicates Ontario Health Insurance Plan.

### Exposure Status

The study exposure was mode of conception as recorded in BORN, namely (1) unassisted conception (reference group); (2) subfertility, defined as a history of an infertility consultation with a physician within 2 years before conception, identified by Ontario Health Insurance Plan (OHIP) billing code 628 from the *International Classification of Diseases, Ninth Revision (ICD-9)* in the absence of fertility treatment, (3) OI or IUI; and (4) IVF or ICSI.^29,30,37,38^

### Outcome

The main outcome of interest was a diagnosis of ASD in the child, starting at age 18 months. A diagnosis of ASD was based on 2 or more outpatient diagnoses (OHIP billing code *ICD-9* 299) identified by either a pediatrician or psychiatrist, and/or 1 or more diagnoses during a hospitalization (*ICD-10-CA* F84).^39^ A similar definition, using US private health plan data, had a positive predictive value of 87.4%.^40^

### Covariates

We adjusted for potential factors that might confound the association between fertility treatment and risk of ASD, including maternal age, parity, income quintile, rurality, immigration status, smoking, illicit substance use, alcohol use, prepregnancy diabetes or chronic hypertension, obesity, history of mental illness within 2 years before the estimated date of conception and up to 19 months post partum, and a history maternal ASD. Infant sex at birth was also adjusted for.

### Statistical Analysis

First, using a conventional modeling approach (eFigure 1 in Supplement 1), time-to-event analyses were conducted using multivariable Cox regression models to estimate hazard ratios (HRs) and 95% CIs, with the child’s age as the underlying time scale, starting at age 18 months (ie, time zero). A robust sandwich-type estimator was used to account for the potential of more than 1 birth to the same woman across the study period. Censoring was at death, loss of OHIP eligibility, or arrival at the end of the study period of June 30, 2022. The number of person-years between the earliest of the ASD diagnosis date or the censoring date, and the birth date was calculated using the SAS yrdif() function. In addition, calculated adjusted absolute rate differences (aARD) reflected the number of excess cases of ASD per 1000 person-years associated with each study exposure, relative to unassisted pregnancy (the referent).

#### Causal Mediation Analysis

Next, we performed causal mediation analysis to describe the mediating role of 6 adverse pregnancy outcomes that have been reported to be associated with infertility and fertility treatments: preeclampsia, cesarean delivery, multifetal pregnancy, preterm birth at less than 37 weeks’ gestation, and severe neonatal morbidity (eFigure 2 in Supplement 1).^28,29,30,31,32,41^ Causal mediation analysis is based on a counterfactual framework^42^ and used herein to disentangle the association between mode of conception and ASD (ie, total effect) into the natural direct effect (ie, the association between each mode of conception and ASD in the absence of the mediator) and the natural indirect effect (ie, the association operating through each of the 6 respective mediators mentioned above). We then estimated the proportion of the total effect between mode of conception and ASD mediated through each of the 6 adverse pregnancy outcomes (eFigure 2 in Supplement 1).

#### Additional Analyses

Additional analyses examined the association between mode of conception and ASD, as follows: (1) restricted to singleton births, (2) restricted to women aged less than 45 years, and (3) further adjusting for calendar year. For the mediation analysis, (1) the population was restricted to singleton births, and (2) the reference group for mode of conception was changed from unassisted conception to subfertility.

Statistical significance was set at a 2-sided *P* value of less than .05. All statistical analyses were performed using SAS version 9.4 for UNIX (SAS Institute Inc). Data were analyzed from October 2022 to October 2023.

## Results

Included were 1 370 152 children born to 895 917 mothers who met the study inclusion criteria (Figure). Of these, 1 185 024 (86.5%) were of pregnancies with unassisted conception; 141 180 (10.3%) in individuals with subfertility; 20 429 (1.5%) following OI or IUI; and 23 519 (1.7%) following IVF or ICSI. In contrast to unassisted conception, individuals with subfertility or those receiving fertility treatment were more likely to be older, nulliparous, reside in a higher-income and urban area, and have higher rates of prepregnancy diabetes and chronic hypertension; the mean (SD) age of each group was as follows: 30.1 (5.2) years in the unassisted conception group, 33.3 (4.7) years in the subfertility group, 33.1 (4.4) years in the OI or IUI group, and 35.8 (4.9) years in the IVF or ICSI group (Table 1).

**Table 1.** Characteristics of 1 370 152 Live-Born Children in Ontario, 2006 to 2018, According to Exposure Group

Characteristic	Participants, No. (%)			
	Unassisted conception (n = 1 185 024)	Subfertility (n = 141 180)	OI\IUI (n = 20 429)	IVF\ICSI (n = 23 519)
Maternal age				
Mean (SD), y	30.1 (5.2)	33.3 (4.7)	33.1 (4.4)	35.8 (4.9)
<35	943 624 (79.7)	84 739 (60.0)	12 873 (63.0)	9853 (41.9)
35-44	239 884 (20.2)	55 561 (39.4)	7488 (36.7)	12 466 (53.0)
45-55	1516 (0.1)	880 (0.6)	68 (0.3)	1200 (5.1)
Income quintile				
1 (Lowest)	258 610 (21.8)	22 124 (15.7)	2558 (12.5)	2316 (9.9)
2	236 974 (20.0)	24 978 (17.7)	3489 (17.1)	3680 (15.7)
3	245 637 (20.7)	29 906 (21.2)	4403 (21.6)	4945 (21.0)
4	250 125 (21.1)	34 451 (24.4)	5461 (26.7)	6292 (26.8)
5 (Highest)	193 678 (16.3)	29 721 (21.1)	4518 (22.1)	6286 (26.7)
Rural residence	98 755 (8.3)	6260 (4.4)	1216 (6.0)	864 (3.7)
Immigrant to Canada	271 813 (22.9)	42 242 (29.9)	4072 (19.9)	6432 (27.4)
Primiparous	475 996 (40.2)	70 679 (50.1)	12 940 (63.3)	15 912 (67.7)
Body mass index ≥30^a^	142 603 (12.0)	18 592 (13.2)	4212 (20.6)	2803 (11.9)
Smoking	117 049 (9.9)	4538 (3.2)	528 (2.6)	303 (1.3)
Substance use^b^	20 315 (1.7)	557 (0.4)	90 (0.4)	69 (0.3)
Alcohol use	2153 (0.2)	104 (0.1)	13 (0.1)	14 (0.1)
Prepregnancy diabetes	18 392 (1.6)	4193 (3.0)	673 (3.3)	626 (2.7)
Chronic hypertension	27 486 (2.3)	5241 (3.7)	827 (4.1)	896 (3.8)
History of mental illness^c^	306 293 (25.9)	37 132 (26.3)	5088 (24.9)	5628 (23.9)
History of polycystic ovary syndrome	7965 (0.7)	5488 (3.9)	1449 (7.1)	699 (3.0)
History of endometriosis	2625 (0.2)	1787 (1.3)	239 (1.2)	625 (2.7)
Multifetal pregnancy	27 997 (2.4)	8090 (5.7)	3934 (19.3)	7553 (32.1)
Sex of the child				
Male	608 491 (51.3)	72 519 (51.4)	10 489 (51.3)	11 908 (50.6)
Female	576 533 (48.7)	68 661 (48.6)	9940 (48.7)	11 611 (49.4)

Abbreviations: OI, ovulation induction; ICSI, intracytoplasmic sperm injection; IUI, intrauterine insemination; IVF, in vitro fertilization.

^a^ Body mass index is calculated as weight in kilograms divided by height in meters squared. Included if recorded in Better Outcomes Registry and Network or an Ontario Health Insurance Plan billing code (*ICD 9*-278) for obesity in 2-year lookback before estimated date of conception.

^b^ Substance use is defined as any marijuana, cocaine, gas/glue, hallucinogens, methadone, narcotics, opioids, and other substance use.

^c^ Maternal mental illness is defined as the presence of 1 or more inpatient visits, 1 or more emergency department visits, or 2 or more outpatient visits with a mental health and addictions–related diagnosis (ie, substance use and addiction disorders, psychotic disorders, and mood and anxiety disorders) within 2 years before estimated date of conception and up to 19 months of age for infant, or history of maternal autism spectrum disorder.

Starting at age 18 months, children were followed up for a median (IQR) of 8.1 (5.1-11.2) years. A total of 22 409 children (1.6%) received an ASD diagnosis, occurring at a mean (SD) age of 3.9 (2.4) years. There were 18 689 children (1.6%) with ASD born to individuals with unassisted conception (mean [SD] age at diagnosis, 3.9 [2.5] years); 2858 (2.0%) with ASD born among individuals with subfertility (3.6 [2.3] years); 404 (2.0%) with ASD whose parent received OI or IUI (3.4 [2.0] years); and 458 (1.9%) with ASD among individuals treated with IVF or ICSI (3.4 [2.2] years).

Among all 1 370 152 live-born children included in the main model, the incidence rate of ASD (per 1000 person-years) was 1.93 among those in the unassisted conception group, 2.49 in the subfertility group, 2.72 after OI or IUI, and 2.71 after IVF or ICSI (Table 2). Relative to the unassisted conception reference group, the aHR for ASD was 1.20 (95% CI, 1.15-1.25) in the subfertility group, 1.21 (95% CI, 1.09-1.34) following OI or IUI, and 1.16 (95% CI, 1.04-1.28) after IVF or ICSI (Table 2). The corresponding adjusted rate differences (per 1000 person-years) were 0.30 (95% CI, 0.22-0.38), 0.36 (95% CI, 0.14-0.57), and 0.22 (95% CI, 0.01-0.44).

**Table 2.** Risk of Autism Spectrum Disorder (ASD) by Mode of Conception

Mode of conception	No. with ASD/No. at risk	Rate of ASD per 1000 person-years	Unadjusted hazard ratio (95% CI)	Adjusted hazard ratio (95% CI)^a^
Analysis among all 1 370 152 live-born children (main model)				
Unassisted conception	18 689/1 185 024	1.93	1 [Reference]	1 [Reference]
Subfertility	2858/141 180	2.49	1.29 (1.24-1.34)	1.20 (1.15-1.25)
Ovulation induction or intrauterine insemination	404/20 429	2.72	1.31 (1.18-1.45)	1.21 (1.09-1.34)
In vitro fertilization or intracytoplasmic sperm injection	458/23 519	2.71	1.29 (1.17-1.43)	1.16 (1.04-1.28)
Analysis limited to 185 128 live-born children of individuals with infertility				
Subfertility	2858/141 180	2.49	1 [Reference]	1 [Reference]
Ovulation induction or intrauterine insemination	404/20 429	2.72	1.01 (0.91-1.12)	1.02 (0.92-1.14)
In vitro fertilization or intracytoplasmic sperm injection	458/23 519	2.71	1.00 (0.90-1.11)	0.94 (0.84-1.05)
Analysis limited to 23 519 live-born children of individuals who underwent in vitro fertilization or intracytoplasmic sperm injection				
In vitro fertilization	408/20 968	2.70	1 [Reference]	1 [Reference]
Intracytoplasmic sperm injection	50/2551	2.77	1.01 (0.75-1.37)	1.05 (0.77-1.42)

^a^ Adjusted for maternal age, parity, income quintile, rurality, immigration status, smoking, obesity, any drug or alcohol use, maternal history of mental illness or ASD, prepregnancy diabetes mellitus or chronic hypertension, and infant sex.

In the analysis limited solely to individuals with infertility, relative to the subfertility group, the aHR for ASD was 1.02 (95% CI, 0.92-1.14) following OI or IUI and 0.94 (95% CI, 0.84-1.05) after IVF or ICSI (Table 2). In another analysis limited to 23 519 live-born children, compared with IVF, those who received ICSI had an aHR for ASD of 1.05 (95% CI, 0.77-1.42) (Table 2).

In additional analysis 1, which was restricted to singleton births, relative to unassisted conception, the corresponding aHRs were comparable with those described above in the main model, including for subfertility (1.20; 95% CI, 1.15-1.25) and OI or IUI (1.15; 95% CI, 1.03-1.29) (eTable 2 in Supplement 1). However, the association between IVF or ICSI and ASD was attenuated (aHR, 1.03; 95% CI, 0.91-1.17). In the additional analysis restricted to mothers aged less than 45 years (eTable 3 in Supplement 1), and further adjusting for calendar year (eTable 4 in Supplement 1), the aHRs were similar to those seen in the main model.

### Causal Mediation Analysis

Selected adverse pregnancy outcomes mediated to different degrees the association between mode of conception and ASD risk (Table 3). For all modes of conception, the proportion mediated by preeclampsia was less than 10% and not statistically significant. In contrast, following OI or IUI, the proportion mediated by cesarean birth was was 11%, by multifetal pregnancy was 36%, by preterm birth was 26%, and by severe neonatal morbidity was 14% (Table 3). After IVF or ICSI, mediation by cesarean birth was 29%, by multifetal pregnancy was 78%, by preterm birth was 50%, and by severe neonatal morbidity was 25%. In individuals receiving IVF or ICSI, the mediation effect of multiple pregnancy on ASD risk was 78.3%.

**Table 3.** Mediation Analysis of the Effect of Selected Adverse Pregnancy Outcomes on the Association Between Mode of Conception and Autism Spectrum Disorder

Adverse pregnancy outcome mediator assessed and mode of conception^b^	Adjusted hazard ratio (95% CI)^a^			Proportion mediated (%)
	Total effect	Natural direct effect	Natural indirect effect	
Preeclampsia				
Subfertility	1.19 (1.16-1.23)	1.19 (1.17-1.22)	1.00 (0.98-1.02)	1.2
Ovulation induction or intrauterine insemination	1.20 (1.14-1.27)	1.20 (1.14-1.26)	1.01 (0.99-1.03)	4.0
In vitro fertilization or intracytoplasmic sperm injection	1.16 (1.10-1.22)	1.14 (1.09-1.20)	1.01 (0.99-1.03)	8.7
Cesarean birth				
Subfertility	1.20 (1.16-1.23)	1.18 (1.16-1.21)	1.01 (0.99-1.03)	7.4
Ovulation induction or intrauterine insemination	1.21 (1.14-1.27)	1.19 (1.13-1.25)	1.02 (1.00-1.04)	10.6
In vitro fertilization or intracytoplasmic sperm injection	1.14 (1.09-1.20)	1.10 (1.05-1.16)	1.04 (1.02-1.06)	28.9^c^
Planned cesarean birth^d^				
Subfertility	1.18 (1.14-1.21)	1.16 (1.14-1.19)	1.01 (0.99-1.03)	7.1
Ovulation induction or intrauterine insemination	1.18 (1.11-1.26)	1.16 (1.09-1.23)	1.02 (1.00-1.04)	12.0
In vitro fertilization or intracytoplasmic sperm injection	1.12 (1.05-1.20)	1.08 (1.02-1.15)	1.04 (1.02-1.06)	34.7^c^
Unplanned Caesarian birth^e^				
Subfertility	1.19 (1.16-1.23)	1.19 (1.16-1.21)	1.01 (0.99-1.03)	4.2
Ovulation induction or intrauterine insemination	1.21 (1.14-1.29)	1.20 (1.13-1.27)	1.01 (0.99-1.03)	5.8
In vitro fertilization or intracytoplasmic sperm injection	1.12 (1.05-1.19)	1.09 (1.03-1.15)	1.02 (1.00-1.05)	22.7^c^
Multiple pregnancy				
Subfertility	1.17 (1.13-1.21)	1.15 (1.12-1.18)	1.01 (0.99-1.03)	8.5
Ovulation induction or intrauterine insemination	1.20 (1.13-1.27)	1.13 (1.07-1.19)	1.06 (1.04-1.09)	35.8^c^
In vitro fertilization or intracytoplasmic sperm injection	1.14 (1.08-1.21)	1.03 (0.98-1.09)	1.11 (1.08-1.14)	78.3^c^
Preterm birth <37 wk				
Subfertility	1.19 (1.16-1.23)	1.17 (1.15-1.20)	1.02 (1.00-1.03)	9.2
Ovulation induction or intrauterine insemination	1.19 (1.13-1.26)	1.14 (1.09-1.20)	1.04 (1.02-1.06)	25.6^c^
In vitro fertilization or intracytoplasmic sperm injection	1.16 (1.10-1.23)	1.08 (1.03-1.14)	1.07 (1.05-1.10)	49.8^c^
Severe neonatal morbidity				
Subfertility	1.20 (1.16-1.23)	1.19 (1.16-1.21)	1.01 (0.99-1.03)	5.1
Ovulation induction or intrauterine insemination	1.20 (1.14-1.27)	1.17 (1.11-1.23)	1.02 (1.00-1.04)	13.9^c^
In vitro fertilization or intracytoplasmic sperm injection	1.16 (1.10-1.22)	1.12 (1.07-1.18)	1.04 (1.02-1.06)	25.0^c^

^a^ Adjusted for maternal age, parity, income quintile, rurality, immigration status, smoking, obesity, any drug or alcohol use, maternal history of mental illness or autism spectrum disorder, prepregnancy diabetes or chronic hypertension, and infant sex.

^b^ Each mode of conception is compared with a birth following unassisted conception (reference group).

^c^ Statistically significant natural indirect effect at a *P* value of less than .05.

^d^ Among 1 176 963 pregnancies.

^e^ Among 1 159 016 pregnancies.

When the mediation analyses were limited to singleton births following IVF or ICSI, mediation by cesarean birth was 69% (eTable 5 in Supplement 1). After changing the mode of conception reference group to births of individuals with subfertility, the mediating effect of preeclampsia was less than 7% for all modes of conception (eTable 6 in Supplement 1). In contrast, after IVF or ICSI, the mediation effect from planned cesarean delivery was 57.1%, and from severe neonatal morbidity, it was 88% (eTable 6 in Supplement 1).

## Discussion

This population-based cohort study observed a slightly higher risk of ASD in children born to an individual with subfertility or those receiving fertility treatment, which appeared to be partly mediated by certain adverse pregnancy factors. Nonetheless, relative to a child born to an individual with subfertility, those conceived by OI or IUI or IVF or ICSI were not associated with a higher risk of ASD. Moreover, when compared with IVF, ICSI was not associated with a higher risk of ASD.

These findings are consistent with prior studies that observed a slightly higher risk of ASD in children born to an individual with isolated infertility or whose parent received fertility treatment.^22,43^ Although infertility treatment was originally thought to be associated with adverse perinatal outcomes^28,31,44,45,46^ increasing evidence suggests that the baseline infertility diagnosis itself may be on the causal pathway.^29,30,32,38^ Similarly, certain adverse pregnancy outcomes are associated with ASD (eg, cesarean birth and preterm birth)^2,3,4,5^ (eFigure 2 in Supplement 1). The US MOSART^47^ study did not find an association between infertility therapy and ASD or any mediation effect by preterm birth. Their study was restricted to singleton pregnancies, which could partly explain some differences from our main results. However, in our additional analysis restricted to singletons, those with subfertility, OI, IUI continued to have an increased risk of ASD compared with unassisted conception.

Mediation analysis performed in the current study suggested that individual adverse pregnancy outcomes (ie, cesarean birth, multiple pregnancy, preterm birth, and severe neonatal morbidity), but not preeclampsia, are associated with ASD risk, especially after IVF or ICSI. For example, in individuals receiving IVF or ICSI, the mediation effect of multiple pregnancy on ASD risk was 78.3%, and the natural direct effect of IVF or ICSI on ASD was markedly attenuated. This would support the continuation of ongoing efforts aimed at minimizing the chances of a multiple pregnancy in patients offered IVF or ICSI.^45,48^ Furthermore, early care plans for pregnant patients following fertility treatment should be established, and strategies to decrease adverse pregnancy outcomes in this population should be implemented.^49^

When studying the association between infertility treatment and adverse maternal and child outcomes, it is essential to contrast the effect measures against individuals with subfertility and who are otherwise not receiving fertility treatment. Our results show that OI or IUI and IVF or ICSI do not appear to introduce any measurable risk of ASD compared with having subfertility alone; rather, underlying infertility might be the driver between parental infertility and ASD in the child, and not the fertility treatments themselves. For example, PCOS, a prevalent metabolic condition often complicated by anovulation and infertility, has been associated with increased odds of ASD in the child.^10,11^ Other metabolic conditions associated with infertility, including obesity^50^ and type 2 diabetes,^51^ are also consistently associated with ASD.^8^

When we focused on the type of fertility therapy offered, ICSI was not associated with a higher risk of ASD compared with IVF. In a Swedish national health registry study comprising a smaller sample of children born following ICSI, the relative risk (RR) of ASD was 4.60 (95% CI, 2.14-9.88) when compared with IVF, but the RR fell to 0.95 (95% CI, 0.13-7.09) when the analysis was restricted to singletons.^20^ In a California study^21^ comparing 21 728 children born after ICSI to 13 753 children born after IVF, the associated aHR for ASD was 1.65 (95% CI, 1.08–2.52) in singleton pregnancies and 1.71 (95% CI, 1.10–2.66) in multiple pregnancies. In that study, the association with ASD was greater when ICSI was used without male factor infertility and with a nonsurgical method of semen collection.^21^ Certainly, diverse techniques and indications for ICSI could explain differences in effect sizes when compared with those seen in the current study.

### Strengths and Limitations

Study strengths include its population-based approach, comprising validated data sets representative of more than 99% of live births in Ontario. One study limitation was the possibility of exposure misclassification. For example, some individuals in the infertility group may have used an OI medication, but could not be identified within the OI or IUI exposure group. Another limitation is the absence of information about the specific reason for infertility (eg, PCOS, endometriosis, tubal factor, male factor), family composition (heterosexual couples, same-sex couples, or single parents by choice), donor oocyte or sperm, or the IVF procedure used (eg, ejaculated vs surgically extracted sperm, fresh vs frozen embryo transfer, or use of preimplantation genetic testing), all of which should be addressed in future studies.

Underestimation of ASD diagnosis using the current data sets has been reported.^52^ This could be problematic if individuals with subfertility, or those in receipt of fertility treatment, were more likely to seek consultation about a neurodevelopmental disorder in their child. There is also the potential for residual confounding, as some variables are not available within the administrative data sets, such as breastfeeding practices,^53^ as well as paternal information, including age.^54,55^ Another limitation is that our mediation analysis method only permitted us to examine each mediator in isolation, even though multiple mediators can co-occur in the same pregnancy. In such cases, it is possible that the mediating effect of a mediator that occurs early in the pregnancy (eg, multiple pregnancy) could itself be mediated by a mediator that occurs subsequently (eg, cesarean delivery). For this reason, the mediated proportion of each mediator cannot be summed to a unified value that represents the combined effect of all mediators.

## Conclusions

In this cohort study of 1.3 million children, there was a slightly higher risk of ASD in those born to an individual with infertility independent of fertility treatment, which appeared partly mediated by certain adverse pregnancy outcomes. Efforts to decrease multifetal pregnancy following OI or IUI and IVF should continue to be reinforced, alongside the development of focused care pregnancy plans both for individuals with subfertility and those receiving fertility treatment. Certainly, major efforts are needed to decrease adverse pregnancy outcomes and optimize neurodevelopment in early childhood.

## Notes

Supplement 1. **eTable 1. **List of Cohort Creation Variable, Study Exposure and Outcome Variables, and Model Covariates **eTable 2. **Risk of Autism Spectrum Disorder (ASD) by Mode of Conception **eTable 3. **Risk of Autism Spectrum Disorder (ASD) by Mode of Conception **eTable 4.** Risk of Autism Spectrum Disorder (ASD) by Mode of Conception **eTable 5. **Mediation Analysis of the Effect of Selected Adverse Pregnancy Outcomes on the Association Between Mode of Conception and Associated Risk of Autism Spectrum Disorder (ASD) **eTable 6. **Mediation Analysis of the Effect of Selected Adverse Pregnancy Outcomes on the Association Between Mode of Conception and Associated Risk of Autism Spectrum Disorder (ASD) Among 1,370,152 Live-Born Children **eFigure 1.** Conventional Model to Examine the Relation Between Mode of Conception and Autism Spectrum Disorder (ASD) in Childhood **eFigure 2. **Causal Mediation Analysis of the Relation Between Mode of Conception and Autism Spectrum Disorder (ASD) in Children

Supplement 2.
Data Sharing Statement
