# Supplementary material for: Infertility and Risk of Autism Spectrum Disorder in Children
Source: JAMA Netw Open. 2023 Nov 20;6(11):e2343954. doi: 10.1001/jamanetworkopen.2023.43954 (PMC10660172; doi:10.1001/jamanetworkopen.2023.43954)
Supplement: Supplement 1. — eTable 1. List of Cohort Creation Variable, Study Exposure and Outcome Variables, and Model Covariates eTable 2. Risk of Autism Spectrum Disorder (ASD) by Mode of Conception eTable 3. Risk of Autism Spectrum Disorder (ASD) by Mode of Conception eTable 4. Risk of Autism Spectrum Disorder (ASD) by Mode of Conception eTable 5. Mediation Analysis of the Effect of Selected Adverse Pregnancy Outcomes on the Association Between Mode of Conception and Associated Risk of Autism Spectrum Disorder (ASD) eTable 6. Mediation Analysis of the Effect of Selected Adverse Pregnancy Outcomes on the Association Between Mode of Conception and Associated Risk of Autism Spectrum Disorder (ASD) Among 1,370,152 Live-Born Children eFigure 1. Conventional Model to Examine the Relation Between Mode of Conception and Autism Spectrum Disorder (ASD) in Childhood eFigure 2. Causal Mediation Analysis of the Relation Between Mode of Conception and Autism Spectrum Disorder (ASD) in Children [file jamanetwopen-e2343954-s001.pdf]

Supplemental Online Content

Velez MP, Dayan N, Shellenberger J, et al. Infertility and risk of autism spectrum disorder in children. *JAMA Netw Open*. 2023;6(11):e2343954. doi:10.1001/jamanetworkopen.2023.43954

eTable 1. List of Cohort Creation Variable, Study Exposure and Outcome Variables, and Model Covariates

eTable2. Risk of Autism Spectrum Disorder (ASD) by Mode of Conception

eTable 3. Risk of Autism Spectrum Disorder (ASD) by Mode of Conception

eTable 4. Risk of Autism Spectrum Disorder (ASD) by Mode of Conception

eTable 5. Mediation Analysis of the Effect of Selected Adverse Pregnancy Outcomes on the Association Between Mode of Conception and Associated Risk of Autism Spectrum Disorder (ASD)

eTable 6. Mediation Analysis of the Effect of Selected Adverse Pregnancy Outcomes on the Association Between Mode of Conception and Associated Risk of Autism Spectrum Disorder (ASD) Among 1,370,152 Live-Born Children

eFigure 1. Conventional Model to Examine the Relation Between Mode of Conception and Autism Spectrum Disorder (ASD) in Childhood

eFigure 2. Causal Mediation Analysis of the Relation Between Mode of Conception and Autism Spectrum Disorder (ASD) in Children

This supplemental material has been provided by the authors to give readers additional information about their work.

© 2023 Velez MP et al. *JAMA Network Open*.

eTable 1. List of Cohort Creation Variable, Study Exposure and Outcome Variables, and Model Covariates.

Assessment	Timing	Disease, Procedure, or Condition	CIHI-DAD, SDS or NACRS ICD-10-CA diagnosis or CCI procedure codes	OHIP ICD-9 diagnosis or fee codes; or other data source	Hyperlink to a validation study or documentation for related codes
Inclusion criteria	April 1, 2006 – March 31, 2015	Live birth record in BORN		BORN	
Exclusion criteria	Same as above	Gestational age < 24 weeks		BORN BIS: ga_at_birth_weeks<br><br>NIDAY: gest	
	Same as above	Maternal age < 18 or > 55 years		BORN BIS: matageatstillorlivebirthyears When maternal birthdate was missing in BORN, the birthdate found in RPDB was used instead<br><br>NIDAY: m_bdate	
	Same as above	Induction for pregnancy termination		BORN: ind_for_lbr_induct_prim_id = 1014250 or all_ind_for_lbr_induct_id = 1014250	
	Same as above	Surrogacy pregnancy		BORN: conception_type_id = 1013170	
	April 1, 2004 – March 31, 2015	< 2 years of maternal OHIP eligibility prior to birth		RPDB	
	April 1, 2006 – September 30, 2016	Child death before age 18 months of age		RPDB	
	April 1, 2006 – March 31, 2015	Pregnancies delivered outside of Ontario		BORN: lbm_submitted	
	Same as above	Pregnancies with no infant record		BORN BIS: birth_id_enc<br>NIDAY: b_key	
	Same as above	Invalid or missing maternal or baby IKN		BORN: b_valikn	
	Same as above	Warning in NIDAY		BORN NIDAY: warn= B, C, K, O, P, R	
	Same as above	Pregnancies with year of birth 1900		BORN: b_bdate	
Study exposures	April 1, 2006 – March 31, 2015	Mode of conception:		BORN (see below for component codes)	

© 2023 Velez MP et al. *JAMA Network Open*.

Assessment	Timing	Disease, Procedure, or Condition	CIHI-DAD, SDS or NACRS ICD-10-CA diagnosis or CCI procedure codes	OHIP ICD-9 diagnosis or fee codes; or other data source	Hyperlink to a validation study or documentation for related codes
	Same as above	Spontaneous conception (unassisted)		No history of infertility (OHIP ICD-9 = 628), and one of the following:<br><br>BORN BIS: CONCEPTION_TYPE_ID = 1013160, 1013180<br><br>OR<br><br>NIDAY: REPASS = 1,9,0	
	Same as above	Subfertility (infertility without fertility treatment)		History of infertility (OHIP ICD-9=628), and one of the following:<br><br>BORN BIS: CONCEPTION_TYPE_ID = 1013160, 1013180<br><br>OR<br><br>NIDAY: REPASS = 1,9,0	
	Same as above	Invasive infertility treatment (In-vitro fertilization / intra-cytoplasmic sperm injection)		BORN BIS: CONCEPTION_TYPE_ID = 1013130, 1013140<br><br>NIDAY: REPASS = 3,4	
	Same as above	Non-invasive infertility treatment (Ovulation induction, intrauterine insemination)		BORN BIS: CONCEPTION_TYPE_ID = 1013110, 1013120, 1013150, 3000006<br><br>NIDAY: REPASS = 2,5	
Main study outcome	April 1, 2006 – March 31, 2022	Autism Spectrum Disorder	ICD-10-CA(DAD): F84	OHIP ICD-9: 299	https://doi.org/10.1001/jama.2017.3415<br>https://doi.org/10.1177/1362361312467709
Mediators	April 1, 2006 – March 31, 2015	Planned Caesarean delivery		BORN BIS: cs_type_id = 1013360, 1013370, 1048727<br><br>NIDAY: deltype=2 and cstype=1	

© 2023 Velez MP et al. *JAMA Network Open*.

Assessment	Timing	Disease, Procedure, or Condition	CIHI-DAD, SDS or NACRS ICD-10-CA diagnosis or CCI procedure codes	OHIP ICD-9 diagnosis or fee codes; or other data source	Hyperlink to a validation study or documentation for related codes
	Same as above	Unplanned Caesarean delivery		BORN BIS: cs_type_id = 1013380<br><br>NIDAY: deltype=2 and cstype=2	
	Same as above	Preterm birth	ICD-10-CA (DAD,SDS): P05, P07, O60	BORN<br>BORN was the primary source for gestational age. When missing, gestational age was imputed using the algorithm from Margulis et al.	https://doi.org/10.1002/pds.3284
	Same as above	Multiple pregnancy		BORN BIS: number_of_fetuses >1<br><br>NIDAY: multgest >1	

© 2023 Velez MP et al. *JAMA Network Open*.

	April 1, 2005 – March 31, 2019	Severe neonatal morbidity	ICD-10-CA (DAD): I63, P100-P103, P130, P132, P133, P140, P141, P220, P23, P271, P280, P285, P36, P521, P522, P77, P90, P912, P915, P916, P9181, R56<br><br>CCI (DAD): 1AA52, 1AA87, 1AC87, 1AE87, 1AF87, 1AG87, 1AJ87, 1AK87, 1AN52, 1AN59, 1AN87, 1AP59, 1AP72, 1AP87, 1AW59, 1AW72, 1AW87, 1AX87, 1BA72, 1BA80, 1BA87, 1BB72, 1BB80, 1BB87, 1BD72, 1BD80, 1BD87, 1BF80, 1BG72, 1BG80, 1BG87, 1BK59, 1BM72, 1BM80, 1BM87, 1BN72, 1BN80, 1BN87, 1BP72, 1BP80, 1BP87, 1BQ72, 1BQ80, 1BQ87, 1BS72, 1BS80, 1BS87, 1BT72, 1BT80, 1BT87, 1GA87, 1GA89, 1GB87, 1GB89, 1GD89, 1GE80, 1GE87, 1GE89, 1GE91, 1GH84, 1GJ86, 1GJ87, 1GK87, 1GK89, 1GM80, 1GM86, 1GM87, 1GN92, 1GR87, 1GR89, 1GR91, 1GT78, 1GT87, 1GT89, 1GT91, 1GV87, 1GV89, 1GW87, 1GX80, 1GX86, 1GX87, 1GY70, 1GY72, 1GY86, 1HJ76, 1HJ82, 1HN87, 1HP76, 1HP78, 1HP80, 1HP82, 1HP83, 1HP87, 1HR80, 1HR84, 1HR87, 1HS80 (excl. 1HS80G),		https://doi.org/10.1007/s10995-016-2047-4

© 2023 Velez MP et al. *JAMA Network Open*.

			1HS90, 1HT80 (excl. 1HT80G), 1HT89, 1HT90, 1HU80 (excl. 1HU80G), 1HU90, 1HV80 (excl. 1HV80G), 1HV90, 1HW78, 1HW79, 1HX80, 1HX87, 1HX80, 1HZ87, 1IA76, 1IA80, 1IA87, 1IB76, 1IB79, 1IB80, 1IB82, 1IB87, 1IC76, 1IC80, 1IC82, 1IC87, 1ID76, 1ID80, 1ID82, 1ID86, 1ID87, 1IF83, 1IJ76, 1IJ80, 1IM76, 1IM80, 1IM82, 1IM83, 1IM87, 1IN83, 1IN84, 1IN87, 1JE57 (excl. 1JE57G), 1JE76, 1JE80, 1JE87, 1JJ76, 1JJ80, 1JK76, 1JK80, 1JK87, 1JW51 (excL. 1JW51G), 1JW57, 1JW76, 1LA84, 1LC84, 1LD84, 1NA72, 1NA74, 1NA76, 1NA77, 1NA80, 1NA84, 1NA86, 1NA87, 1NA88, 1NA89, 1NA90, 1NA91, 1NA92, 1NE80, 1NF76, 1NF78, 1NF80, 1NF82, 1NF84, 1NF86, 1NF87 (excl. 1NF87B), 1NF89, 1NF90, 1NF91, 1NF92, 1NK76, 1NK77, 1NK80, 1NK82, 1NK84, 1NK87 (excl. 1NK87B), 1NM74, 1NM76, 1NM77, 1NM80, 1NM82, 1NM87 (excl. 1NM87B), 1NM89, 1NM91, 1NP72, 1NP73, 1NP86, 1NQ74 (excl. 1NQ74B), 1NQ80,		

© 2023 Velez MP et al. *JAMA Network Open*.

			1NQ84, 1NQ86, 1NQ87 (excl. 1NQ87B), 1NQ89, 1NQ90, 1NT80, 1NT84, 1NT86, 1NT87, 1NV89, 1OA87, 1OB87, 1OB89, 1OD76, 1OD89, 1OE76, 1OE80, 1OE89, 1OJ76 (excl. 1OJ76B), 1OJ87, 1OJ89, 1OK87, 1OK89, 1OK91, 1OT72, 1OT87, 1OT91, 1PB87, 1PB89, 1PC80, 1PC87 (excl. 1PC87D), 1PC89, 1PC91, 1PE57 (excl. 1PE57BD), 1PE80 (excl. 1PE80D), 1PE82, 1PE87 (excl. 1PE87D), 1PE89 (excl. 1PE89D), 1PG76, 1PG77, 1PG80 (excl. 1PG80D), 1PG86, 1PG89, 1PL74 (excl. 1PL74CD), 1PL80, 1PM79, 1PM86, 1PM87 (excl. 1PM87B), 1PM89, 1PM90, 1PM91, 1PM92, 1QE53, 1QE80, 1QE82, 1QE84, 1QE87, 1QE89, 1QG89, 1QM74, 1QM80, 1QM87, 1QM89, 1QM91, 1QN82, 1QT87, 1QT91, 1RB74, 1RB80, 1RB83, 1RB87, 1RB89,1RD89, 1RF51, 1RF72, 1RF74, 1RF80, 1RF87, 1RF89, 1RM87 (excl. 1RM87B), 1RM89, 1RM91, 1RN87, 1RN89, 1RS74, 1RS80, 1RS86, 1RS87, 1RS89, 1RW87, 1RW88, 1RW91, 1RW92, 1SA74, 1SA75, 1SA80, 1SA89,		

© 2023 Velez MP et al. *JAMA Network Open*.

			1SC74, 1SC75, 1SC80, 1SC87, 1SC89, 1SE53, 1SE89 (excl. 1SE89D), 1SF80, 1SF87, 1SF89, 1SG80, 1SG87, 1SH87, 1SM74, 1SM80, 1SM87, 1SN87, 1SN93, 1SQ53, 1SQ74, 1SQ80, 1SQ87, 1SQ91, 1SQ93, 1SW74, 1SY80, 1SY84, 1SY87, 1SZ87, 1VA53, 1VA74, 1VA75, 1VA80, 1VA87, 1VA93, 1VC74, 1VC80, 1VC87, 1VC91, 1VC93, 1VE80, 1VG53, 1VG55, 1VG72, 1VG73, 1VG74, 1VG75, 1VG80, 1VG87, 1VG93, 1VK80, 1VK87, 1VK89, 1VL80, 1VL87, 1VM80, 1VM87, 1VN80, 1VN87, 1VP74, 1VP80, 1VP87, 1VP89, 1VQ74, 1VQ79, 1VQ80, 1VQ82, 1VQ87, 1VQ91, 1VQ93, 1VS72, 1VS80, 1VX87, 1LZ35CAE6, 1LZ35HAC1, 1LZ35HAC5, 1LZ35HAC6, 1LZ35HAC7, 1LZ35HAE6, 1LZ35HAT7, 1LZ35HAT9, 1LZ35HAZ9, 1LZ35HHC1, 1LZ35HHC5, 1LZ35HHC6, 1LZ35HHC7, 1LZ35HHE0, 1LZ35HHE6, 1LZ35HHT7, 1LZ35HHT9,		

© 2023 Velez MP et al. *JAMA Network Open*.

			1LZ35HHZ9, 1LZ35HRC5, 1LZ35HRC6, 1LZ35HRC7, 1LZ35HRT9, 1LZ35HRZ9, 1KV53HACH, 1KV53HAFT, 1KV53LAFT, 2IM28GP, 2LZ28GQPL, 2LZ28GRPL, 2LZ28JAPL, 1KX53HACH, 1KX53HAFT, 1KX53 LAFT, 2LZ28GQPL, 2LZ28GRPL, 1GV52DA, 1GV52DATS, 1GV52HA, 1GV52HAHE, 1GV52HATK, 1GV52LA, 1GV52LATS, 1GV52LAXXE, 1GV54JATS, 1GV55JATS, 1HZ30JN, 1HZ30JY, 1GZ30CJ, 1GZ30CJNB, 1GZ30JH, 1LZ19HHU1A, 1LZ19HHU1J, 1LZ19HHU2A, 1LZ19HHU2J, 1LZ19HHU3J, 1LZ19HHU4J, 1LZ19HHU5J, 1LZ19HHU6A, 1LZ19HHU6J, 1LZ19HHU9A, 1LZ19HHU9J, 1LZ19HMU1, 1LZ19HMU2, 1LZ19HMU9, 1LZ35HAC5, 1GZ31CAEP, 1GZ31CAND, 1GZ31CAPK,		

© 2023 Velez MP et al. *JAMA Network Open*.

Assessment	Timing	Disease, Procedure, or Condition	CIHI-DAD, SDS or NACRS ICD-10-CA diagnosis or CCI procedure codes	OHIP ICD-9 diagnosis or fee codes; or other data source	Hyperlink to a validation study or documentation for related codes
			1GZ31CBND, 1GZ31CRND, 1GZ31GPND, 1GZ31JAGX, 1GZ31JAMD, 1GZ31JANC, 1GZ31JAPK		
	April 1, 2006 – March 31, 2015	Preeclampsia	ICD-10-CA (DAD,SDS,NACRS): O11, O13, O14, O15		
Covariates	April 1, 2006 – March 31, 2015	Maternal age		RPDB<br>BORN	
	Same as above	Gestational age	ICD-10-CA (DAD,SDS): P05, P07, O60	BORN<br>BORN was the primary source for gestational age. When missing, gestational age was imputed using the algorithm from Margulis et al.	https://doi.org/10.1002/pds.3284
	Same as above	Baby sex		RPDB	
	Same as above	Maternal smoking history		BORN BIS: mat_smoking_at_adm_for_birth_id = 1017390, 1017400, 1017410, 1017420<br>NIDAY: smoking = 3, 4	
	Same as above	Maternal drug use history		BORN BIS: expos_drug_and_subst_id = 1020460, 1020470, 1020480, 1020490, 1020500, 1020510, 1020520, 1020530<br>NIDAY: value of 1 in mathp6, mathp7, mathp8, mathp9, mathp10, mathp11, mathp13	
	Same as above	Maternal alcohol history		BORN BIS: preg_expos_alcohol_id = 1020430, 1020440, 1020442<br>NIDAY: mathp1 = 1	

© 2023 Velez MP et al. *JAMA Network Open*.

Assessment	Timing	Disease, Procedure, or Condition	CIHI-DAD, SDS or NACRS ICD-10-CA diagnosis or CCI procedure codes	OHIP ICD-9 diagnosis or fee codes; or other data source	Hyperlink to a validation study or documentation for related codes
	April 1, 2003 – April 30, 2016	Maternal mental health diagnosis history	ICD-10-CA (DAD, NACRS):<br>F06-F09, F1-F6, F80, F81 F92-F95, F98	OHIP:<br>location = O, L, or H, AND<br>spec = 00 or 19, AND<br>ICD-9 295-298, 300-304, 306, 309, 311<br><br>DSM-4 (OMHRS): 291-293, 295-298, 300, 3000-301,0 30113, 3012-3019, 3022-3024, 3026-3029, 303-305, 30651, 307, 3070-3079, 3083, 309, 3090, 3092- 3094, 3099, 310, 31,1 315, 316, 327, 331-333, 347, 394, 395, 403, 648, 652, 780, 799, 967, 995, V15, V40, V61, V62, V65, V70, V71	https://doi.org/10.1097/00005650-200410000-00004<br>(Steele algorithm where the OHIP codes are from)<br><br>https://www.ices.on.ca/Publications/Atlases-and-Reports/2018/MHASEF<br>(Source for DSM-4 and ICD-10-CA codes, as per concept dictionary)
	Same as above	Maternal ASD diagnosis history	ICD-10-CA(DAD): F84	OHIP dxcode: 299	
	April 1, 2006 – March 31, 2015	Income quintile		RPDB	
	Same as above	Rurality		RPDB	
	Same as above	Immigrant status		Immigration Refugees and Citizenship Canada Permanent Resident (IRCC-PR) Database	
	Same as above	BMI		BORN BIS: pre_preg_maternal_weight_kg, maternal_height_cm<br><br>NIDAY: matwgtkg, mathgtm	
	Same as above	Obesity		BORN (BMI > 30)<br>OHIP ICD-9: 278	
	Same as above	Parity		BORN<br>MOMBABY	
	April 1, 1991 – March 31, 2015	Diabetes		ODD	https://doi.org/10.1186/s12913-018-3148-0

© 2023 Velez MP et al. *JAMA Network Open*.

eTable2. Risk of Autism Spectrum Disorder (ASD) by Mode of Conception. These analyses are limited to singleton live births.

Mode of conception	No. with ASD/ No. at risk	Rate of ASD per 1000 person-years	Unadjusted hazard ratio (95% CI)	Adjusted hazard ratio (95% CI)[a]
Analysis among all 1,322,578 singleton live-born children				
Unassisted conception	18,070 / 1,157,027	1.91	1.00 (ref)	1.00 (ref)
Subfertility	2668 / 133,090	2.48	1.29 (1.24-1.35)	1.20 (1.15-1.25)
Ovulation induction or intrauterine insemination	308 / 16,495	2.60	1.25 (1.12-1.40)	1.15 (1.03-1.29)
In vitro fertilization or intracytoplasmic sperm injection	266 / 15,966	2.46	1.14 (1.01-1.29)	1.03 (0.91-1.17)
Analysis limited to 165,551 singleton live-born children among individuals with infertility				
Subfertility	2668 / 133,090	2.48	1.00 (Ref.)	1.00 (Ref.)
Ovulation induction or intrauterine insemination	308 / 16,495	2.60	0.96 (0.85-1.08)	0.98 (0.87-1.10)
In vitro fertilization or intracytoplasmic sperm injection	266 / 15,966	2.46	0.88 (0.77-0.99)	0.84 (0.74-0.96)
Analysis limited to 15,966 live-born children among individuals who underwent *In vitro* fertilization or intracytoplasmic sperm injection				
In vitro fertilization	236 / 14,249	2.45	1.00 (Ref.)	1.00 (Ref.)
Intracytoplasmic sperm injection	30 / 1717	2.60	1.06 (0.72-1.54)	1.09 (0.75-1.59)

[a] Adjusted for maternal age, parity, income quintile, rurality, immigration status, smoking, obesity, any drug or alcohol use, maternal history of mental illness or ASD, pre-pregnancy diabetes mellitus or chronic hypertension, and infant sex.

© 2023 Velez MP et al. *JAMA Network Open*.

eTable 3. Risk of Autism Spectrum Disorder (ASD) by Mode of Conception. These analyses are limited to live-born children of mothers aged < 45 years, including by mode of conception.

Mode of conception	No. with ASD/ No. at risk	Rate of ASD per 1000 person-years	Unadjusted hazard ratio (95% CI)	Adjusted hazard ratio (95% CI)[a]
Analysis among all 1,366,488 live-born children of women aged < 45 years				
Unassisted conception	18,659 / 1,183,508	1.93	1.00 (Referent)	1.00 (Referent)
Subfertility	2838 / 140,300	2.49	1.29 (1.24-1.34)	1.20 (1.15-1.25)
Ovulation induction or intrauterine insemination	404 / 20,361	2.73	1.31 (1.19-1.46)	1.21 (1.09-1.34)
In vitro fertilization or intracytoplasmic sperm injection	426 / 22,319	2.64	1.27 (1.14-1.40)	1.14 (1.03-1.27)
Analysis limited to 182,980 live-born children of individuals aged < 45 years with infertility				
Subfertility	2838 / 140,300	2.49	1.00 (Referent)	1.00 (Referent)
Ovulation induction or intrauterine insemination	404 / 20,361	2.73	1.01 (0.91-1.13)	1.02 (0.92-1.14)
In vitro fertilization or intracytoplasmic sperm injection	426 / 22,319	2.64	0.98 (0.88-1.09)	0.93 (0.83-1.04)
Analysis limited to 22,319 live-born children of individuals aged < 45 years who underwent *In vitro* fertilization or intracytoplasmic sperm injection				
In vitro fertilization	378 / 19,882	2.63	1.00 (Referent)	1.00 (Referent)
Intracytoplasmic sperm injection	48 / 2437	2.76	1.04 (0.76-1.42)	1.06 (0.78-1.45)

[a] Adjusted for maternal age, income quintile, rurality, immigration status, smoking, obesity, parity, any drug or alcohol use, maternal history of mental illness or ASD, pre-pregnancy diabetes mellitus or chronic hypertension, and infant sex.

© 2023 Velez MP et al. *JAMA Network Open*.

eTable 4. Risk of Autism Spectrum Disorder (ASD) by Mode of Conception. Analysis additionally adjusted for calendar year.

Mode of conception	No. with ASD/ No. at risk	Rate of ASD per 1000 person-years	Unadjusted hazard ratio (95% CI)	Adjusted hazard ratio (95% CI)[a]
Analysis among all 1,370,152 singleton live-born children				
Unassisted conception	18,689 / 1,185,024	1.93	1.00 (Referent)	1.00 (Referent)
Subfertility	2858 / 141,180	2.49	1.29 (1.24-1.34)	1.20 (1.15-1.25)
Ovulation induction or intrauterine insemination	404 / 20,429	2.72	1.31 (1.18-1.45)	1.21 (1.09-1.34)
In vitro fertilization or intracytoplasmic sperm injection	458 / 23,519	2.71	1.29 (1.17-1.43)	1.16 (1.04-1.28)
Analysis limited to 185,128 singleton live-born children of individuals with infertility				
Subfertility	2858 / 141,180	2.49	1.00 (Referent)	1.00 (Referent)
Ovulation induction or intrauterine insemination	404 / 20,429	2.72	1.01 (0.91-1.12)	1.03 (0.92-1.15)
In vitro fertilization or intracytoplasmic sperm injection	458 / 23,519	2.71	1.00 (0.90-1.11)	0.95 (0.85-1.05)
Analysis limited to 23,519 live-born children of individuals who underwent i*n vitro* fertilization or intracytoplasmic sperm injection				
In vitro fertilization	408 / 20,968	2.70	1.00 (Referent)	1.00 (Referent)
Intracytoplasmic sperm injection	50 / 2,551	2.77	1.01 (0.75-1.37)	1.05 (0.77-1.42)

[a] Adjusted for maternal age, parity, income quintile, rurality, immigration status, smoking, obesity, any drug or alcohol use, maternal history of mental illness or ASD, pre-pregnancy diabetes mellitus or chronic hypertension, infant sex, and calendar year

© 2023 Velez MP et al. *JAMA Network Open*.

eTable 5. Mediation Analysis of the Effect of Selected Adverse Pregnancy Outcomes on the Association Between Mode of Conception and Associated Risk of Autism Spectrum Disorder (ASD). Each mode of conception is compared to a birth in a mother with unassisted conception (reference group). This analysis is restricted to 1,322,578 singleton live births.

**Adverse pregnancy outcome mediator assessed**	**Mode of conception**	**Adjusted hazard ratio, total effect (95% CI)**	**Adjusted hazard ratio[a], natural direct effect (95% CI)**	**Adjusted hazard ratio[a], natural indirect effect (95% CI)**	**Proportion mediated (%)**
Preeclampsia	Subfertility	1.20 (1.16-1.23)	1.20 (1.17-1.22)	1.00 (0.98-1.02)	0.6
	Ovulation induction or intrauterine insemination	1.15 (1.09-1.22)	1.15 (1.08-1.22)	1.00 (0.98-1.02)	2.8
	In vitro fertilization or intracytoplasmic sperm injection	1.03 (0.97-1.10)	1.03 (0.97-1.09)	1.01 (0.99-1.02)	16.3
Caesarean birth	Subfertility	1.20 (1.16-1.23)	1.19 (1.16-1.21)	1.01 (0.99-1.03)	5.5
	Ovulation induction or intrauterine insemination	1.15 (1.09-1.23)	1.15 (1.08-1.21)	1.01 (0.99-1.03)	5.5
	In vitro fertilization or intracytoplasmic sperm injection	1.03 (0.96-1.10)	1.01 (0.95-1.07)	1.02 (1.00-1.04)	69.0[b]
Planned Caesarean birth[c]	Subfertility	1.18 (1.14-1.21)	1.17 (1.14-1.19)	1.01 (0.99-1.03)	5.7
	Ovulation induction or intrauterine insemination	1.16 (1.08-1.25)	1.15 (1.08-1.23)	1.01 (0.99-1.03)	5.6
	In vitro fertilization or intracytoplasmic sperm injection	1.04 (0.97-1.12)	1.02 (0.95-1.10)	1.02 (1.00-1.04)	49.5
Unplanned Caesarian birth[d]	Subfertility	1.20 (1.16-1.24)	1.19 (1.16-1.22)	1.00 (0.98-1.03)	2.7
	Ovulation induction or intrauterine insemination	1.16 (1.09-1.24)	1.16 (1.09-1.23)	1.00 (0.98-1.02)	2.5
	In vitro fertilization or intracytoplasmic sperm injection	1.01 (0.93-1.08)	0.99 (0.93-1.07)	1.01 (0.99-1.03)	N/A[e]
Preterm birth < 37 weeks	Subfertility	1.20 (1.16-1.23)	1.19 (1.16-1.21)	1.01 (0.99-1.03)	4.1
	Ovulation induction or intrauterine insemination	1.15 (1.09-1.23)	1.15 (1.08-1.21)	1.01 (0.99-1.03)	4.4
	In vitro fertilization or intracytoplasmic sperm injection	1.03 (0.97-1.10)	1.02 (0.96-1.08)	1.02 (1.00-1.04)	48.9
Neonatal adverse morbidity	Subfertility	1.20 (1.16-1.23)	1.19 (1.17-1.22)	1.00 (0.99-1.02)	2.7
	Ovulation induction or intrauterine insemination	1.15 (1.09-1.22)	1.15 (1.08-1.21)	1.00 (0.99-1.02)	3.2
	In vitro fertilization or intracytoplasmic sperm injection	1.03 (0.96-1.10)	1.02 (0.96-1.09)	1.01 (0.99-1.03)	28.9

[a]Adjusted for maternal age, parity, income quintile, rurality, immigration status, smoking, obesity, any drug or alcohol use, maternal history of mental illness or ASD, pre-pregnancy diabetes mellitus or chronic hypertension, and infant sex.

[b]Statistically significant natural indirect effect at a P-value < 0.05.

[c]Among 135,058 pregnancies.

[d]Among 135,139 pregnancies.

© 2023 Velez MP et al. *JAMA Network Open*.

[e]Not applicable, as "the [proportion-mediated measure] is problematic when the natural direct effect and natural indirect effect operate in different directions. One can obtain a proportion mediated much larger than 100%, and the measure is no longer really meaningful. Even more dramatically, if the natural direct and indirect effects are of roughly the same magnitude but of opposite signs, then the total effect will be close to zero and the proportion-mediated measure then takes the natural indirect effect and divides it by a number close to zero which will result in an enormous (and again meaningless) proportion." Source: VanderWeele TJ. Explanation in Causal Inference: Methods for Mediation and Interaction. New York, NY: Oxford University Press, 2015, page 48.

© 2023 Velez MP et al. *JAMA Network Open*.

eTable 6. Mediation Analysis of the Effect of Selected Adverse Pregnancy Outcomes on the Association Between Mode of Conception and Associated Risk of Autism Spectrum Disorder (ASD) Among 1,370,152 Live-Born Children. Each mode of conception is compared to a birth in a mother with subfertility (reference group).

Adverse pregnancy outcome mediator assessed	Mode of conception	Adjusted hazard ratio, total effect (95% CI)	Adjusted hazard ratio[a], natural direct effect (95% CI)	Adjusted hazard ratio[a], natural indirect effect (95% CI)	Proportion mediated (%)
Preeclampsia	Unassisted conception	0.84 (0.81-0.86)	0.84 (0.82-0.85)	1.00 (0.98-1.02)	0.5
	Ovulation induction or intrauterine insemination	0.96 (0.90-1.02)	0.96 (0.90-1.02)	1.00 (0.98-1.02)	6.6
	In vitro fertilization or intracytoplasmic sperm injection	0.86 (0.81-0.92)	0.86 (0.81-0.92)	1.00 (0.99-1.02)	2.5
Caesarean birth	Unassisted conception	0.83 (0.81-0.86)	0.85 (0.83-0.86)	0.99 (0.97-1.01)	6.3
	Ovulation induction or intrauterine insemination	1.01 (0.95-1.07)	1.00 (0.95-1.06)	1.01 (0.99-1.03)	74.3
	In vitro fertilization or intracytoplasmic sperm injection	0.95 (0.90-1.01)	0.93 (0.89-0.98)	1.02 (1.01-1.04)	51.3[b]
Planned Caesarean birth[c]	Unassisted conception	0.85 (0.82-0.88)	0.86 (0.84-0.88)	0.99 (0.97-1.01)	6.1
	Ovulation induction or intrauterine insemination	1.01 (0.94-1.07)	1.00 (0.94-1.06)	1.01 (0.99-1.03)	N/A[d]
	In vitro fertilization or intracytoplasmic sperm injection	0.95 (0.89-1.02)	0.93 (0.87-0.99)	1.03 (1.01-1.05)	57.1[b]
Unplanned Caesarian birth[e]	Unassisted conception	0.84 (0.81-0.86)	0.84 (0.82-0.86)	0.99 (0.97-1.01)	3.5
	Ovulation induction or intrauterine insemination	1.01 (0.95-1.08)	1.01 (0.95-1.07)	1.00 (0.98-1.02)	22.9
	In vitro fertilization or intracytoplasmic sperm injection	0.93 (0.88-1.00)	0.92 (0.87-0.98)	1.02 (1.00-1.04)	24.5
Multiple pregnancy	Unassisted conception	0.86 (0.83-0.88)	0.87 (0.84-0.89)	0.99 (0.97-1.01)	7.4
	Ovulation induction or intrauterine insemination	1.03 (0.96-1.09)	0.98 (0.92-1.03)	1.05 (1.03-1.07)	N/A[d]
	In vitro fertilization or intracytoplasmic sperm injection	0.98 (0.90 -1.04)	0.89 (0.84-0.95)	1.10 (1.06-1.13)	N/A[d]
Preterm birth < 37 weeks	Unassisted conception	0.84 (0.82-0.86)	0.85 (0.83-0.87)	0.99 (0.97-1.00)	7.9
	Ovulation induction or intrauterine insemination	1.00 (0.95-1.06)	0.98 (0.92-1.03)	1.03 (1.01-1.05)	N/A[d]
	In vitro fertilization or intracytoplasmic sperm injection	0.98 (0.92-1.03)	0.92 (0.87-0.97)	1.06 (1.04-1.08)	N/A[d]
Neonatal adverse morbidity	Unassisted conception	0.84 (0.81-0.86)	0.84 (0.83-0.86)	0.99 (0.97-1.01)	4.3
	Ovulation induction or intrauterine insemination	1.00 (0.95-1.06)	0.99 (0.94-1.04)	1.02 (1.00-1.03)	N/A[d]
	In vitro fertilization or intracytoplasmic sperm injection	0.97 (0.92-1.02)	0.95 (0.90-0.99)	1.03 (1.01-1.05)	88.4[b]

[a]Adjusted for maternal age, parity, income quintile, rurality, immigration status, smoking, obesity, any drug or alcohol use, maternal history of mental illness or ASD, pre-pregnancy diabetes mellitus or chronic hypertension, and infant sex.

[b]Statistically significant natural indirect effect at a P-value < 0.05.

© 2023 Velez MP et al. *JAMA Network Open*.

[c]Among 1,176,963 pregnancies.

[e]Not applicable, as “the [proportion-mediated measure] is problematic when the natural direct effect and natural indirect effect operate in different directions. One can obtain a proportion mediated much larger than 100%, and the measure is no longer really meaningful. Even more dramatically, if the natural direct and indirect effects are of roughly the same magnitude but of opposite signs, then the total effect will be close to zero and the proportion-mediated measure then takes the natural indirect effect and divides it by a number close to zero which will result in an enormous (and again meaningless) proportion.” Source: VanderWeele TJ. Explanation in Causal Inference: Methods for Mediation and Interaction. New York, NY: Oxford University Press, 2015, page 48.

[e]Among 1,159,016 pregnancies.

© 2023 Velez MP et al. *JAMA Network Open*.

eFigure 1. Conventional Model to Examine the Relation Between Mode of Conception and Autism Spectrum Disorder (ASD) in Childhood.

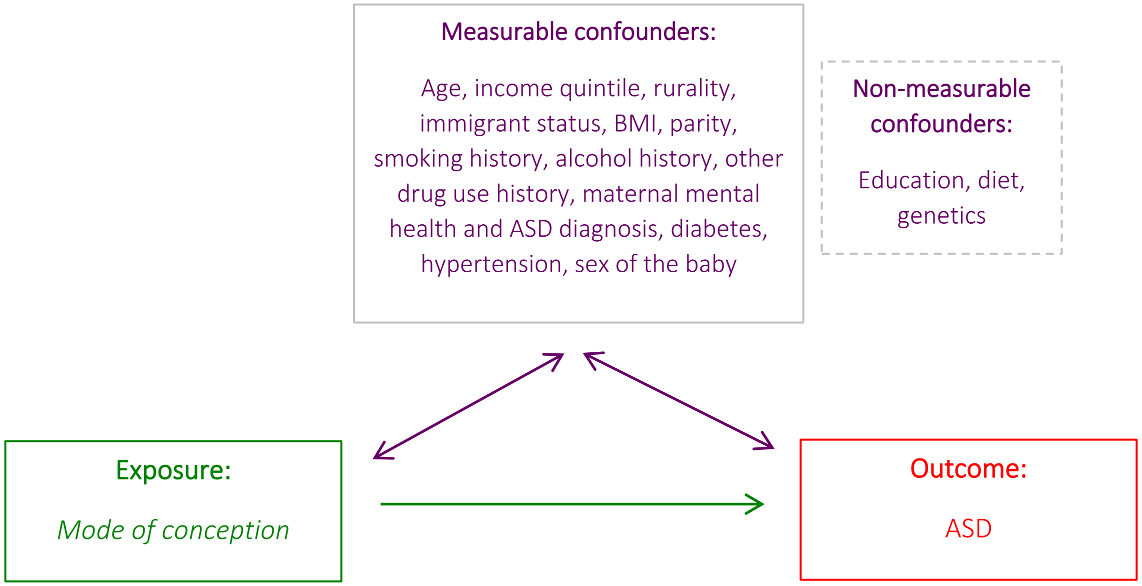

© 2023 Velez MP et al. *JAMA Network Open*.

eFigure 2. Causal Mediation Analysis of the Relation Between Mode of Conception and Autism Spectrum Disorder (ASD) in Children

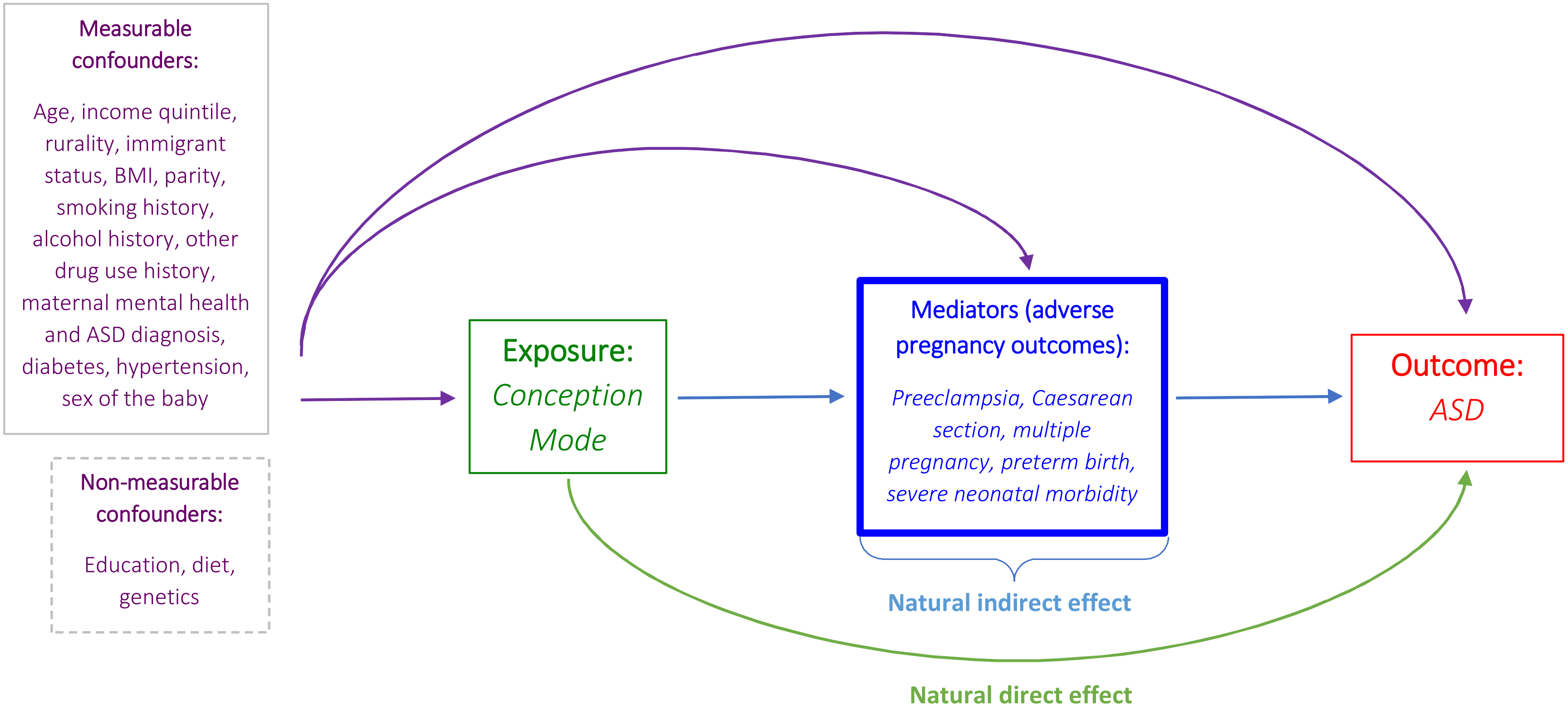

© 2023 Velez MP et al. *JAMA Network Open*.
