# Supplementary material for: Infertility and Risk of Autism Spectrum Disorder in Children
Source: JAMA Netw Open. 2023 Nov 20;6(11):e2343954. doi: 10.1001/jamanetworkopen.2023.43954 (PMC10660172; doi:10.1001/jamanetworkopen.2023.43954)
Supplement: Supplement 2. — Data Sharing Statement [file jamanetwopen-e2343954-s002.pdf]

Data Sharing Statement

Velez. Infertility and Risk of Autism Spectrum Disorder in Children. *JAMA Netw Open*. Published November 20, 2023. doi:10.1001/jamanetworkopen.2023.43954

Data
Data available: No

Additional Information
Explanation for why data not available: The data from this study are held securely in coded form at ICES. Although data-sharing agreements prohibit ICES from making the data set publicly available, access may be granted to those who meet prespecified criteria for confidential access, available at https://www.ices.on.ca/DAS. The full data set creation plan and underlying analytic code are available from the authors upon request, understanding that the computer programs may rely on coding templates or macros that are unique to ICES and therefore either inaccessible or requiring modification.
